# Deep-learning segmentation of the substantia nigra from multiparametric MRI: Application to Parkinson’s disease

**DOI:** 10.1162/IMAG.a.158

**Published:** 2025-09-29

**Authors:** Peder A.G. Lillebostad, Tormund H. Njølstad, Signe Hogstad, Frank Riemer, Simon U. Kverneng, Kjersti E. Stige, Martin Biermann, Mandar Jog, Sagar Buch, E. Mark Haacke, Charalampos Tzoulis, Arvid Lundervold

**Affiliations:** Department of Biomedicine, University of Bergen, Bergen, Norway; Mohn Medical Imaging and Visualization Center, Department of Radiology, Haukeland University Hospital, Bergen, Norway; Neuro-SysMed, Department of Neurology, Haukeland University Hospital, Bergen, Norway; K.G. Jebsen Center for Translational Research in Parkinson’s disease, University of Bergen, Bergen, Norway; Department of Radiology, Haukeland University Hospital, Bergen, Norway; Department of Physics and Technology, University of Bergen, Bergen, Norway; Department of Clinical Medicine, University of Bergen, Bergen, Norway; Department of Neuromedicine and Movement Sciences, Norwegian University of Science and Technology, Trondheim, Norway; Department of Neurology and Clinical Neurophysiology, St Olav’s University Hospital, Trondheim, Norway; Department of Clinical Neurological Sciences, London Health Sciences Centre, Western University, London, ON, Canada; Department of Neurology, Wayne State University, Detroit, MI, United States; Department of Radiology, Wayne State University, Detroit, MI, United States

**Keywords:** substantia nigra, Parkinson’s disease, neuromelanin MRI, multiparametric MRI, domain shift, segmentation

## Abstract

Loss of dopaminergic neurons in the substantia nigra (SN) pars compacta (SNc) is a pathological hallmark of Parkinson’s disease (PD). This is accompanied by a reduction of the dopamine synthesis byproduct neuromelanin (NM), which can be detected *in vivo* with NM-sensitive MRI, showing potential as a biomarker of PD. This relies on delineating the NM-rich region, which is achieved by applying manual or automated methods. Currently, there is a lack of publicly available tools for this task, so we trained a deep neural network intended for publishing, while exploring the effects of incorporating multiparametric MRI for segmenting the NM hyperintensity of the SN. We obtained multiple MRI contrasts, including NM-sensitive magnetization transfer contrast from 109 individuals (87 PD, 22 healthy controls) comprising a Norwegian and a Canadian cohort. The method was further evaluated on 209 MRIs from the Parkinson’s Progressive Markers Initiative (PPMI). We observed that models trained naively on images from a single site tended to perform very poorly when exposed to similar data from different sites, emphasizing the importance of validating on out-of-distribution data. By applying aggressive data augmentation, we could largely attenuate the problem. We also observed a small additional regularizing effect from training the neural network on multiparametric MRIs. Volume and contrast-to-noise ratio (CNR) of the SN hyperintensity to the crus cerebri were used to distinguish patients from controls, with an area under the receiver operating characteristic (AUROC) of 0.863. CNR was found to be a better marker of disease status than volume, and we discuss a potential confusion in discerning the two measures. No contralateral association was observed between the severity of motor symptoms and volume or CNR.

## 1 Introduction

A pathological hallmark of Parkinson’s disease (PD) is the depigmentation and atrophy of the substantia nigra (SN) *pars compacta* (SNc), due to degeneration and death of this region’s dopaminergic neurons (Dickson, 2012). These neurons play a crucial role in motor control via their innervation of the striatum. Dysfunction and loss of the nigrostriatal connections leads to the cardinal motor symptoms of parkinsonism, including bradykinesia and tremor. The integrity of the nigrostriatal pathway can be assessed *in vivo* using radio-labeling of the neurotransmitter or its precursor, for example, positron emission tomography (PET) with [^18^F]FDOPA, or of the dopamine transporter, for example, single photon emission tomography (SPECT) using [^123^I]ioflupane (DatScan). Both methods are highly sensitive in detecting PD and other neurodegenerative diseases affecting the striata, but have the disadvantage of involving ionizing radiation and being comparatively expensive. Furthermore, while excellent in assessing functional integrity of dopaminergic neurotransmission at the level of the striata, these approaches are not directly informative about the integrity of the neurons in the SNc itself. For these reasons, there is considerable interest in developing MRI-based methods for assessing SNc integrity (Lehéricy et al., 2014). Notable advances in this area include sequences sensitive to neuromelanin content, whose signal and volume are reduced in PD, iron-sensitive sequences detecting increased iron signal as well as topographic alteration (e.g., loss of the nigrosome-1 sign) (Ravanfar et al., 2021), and potentially diffusion-related changes (Deng et al., 2018).

**Fig. 1.**
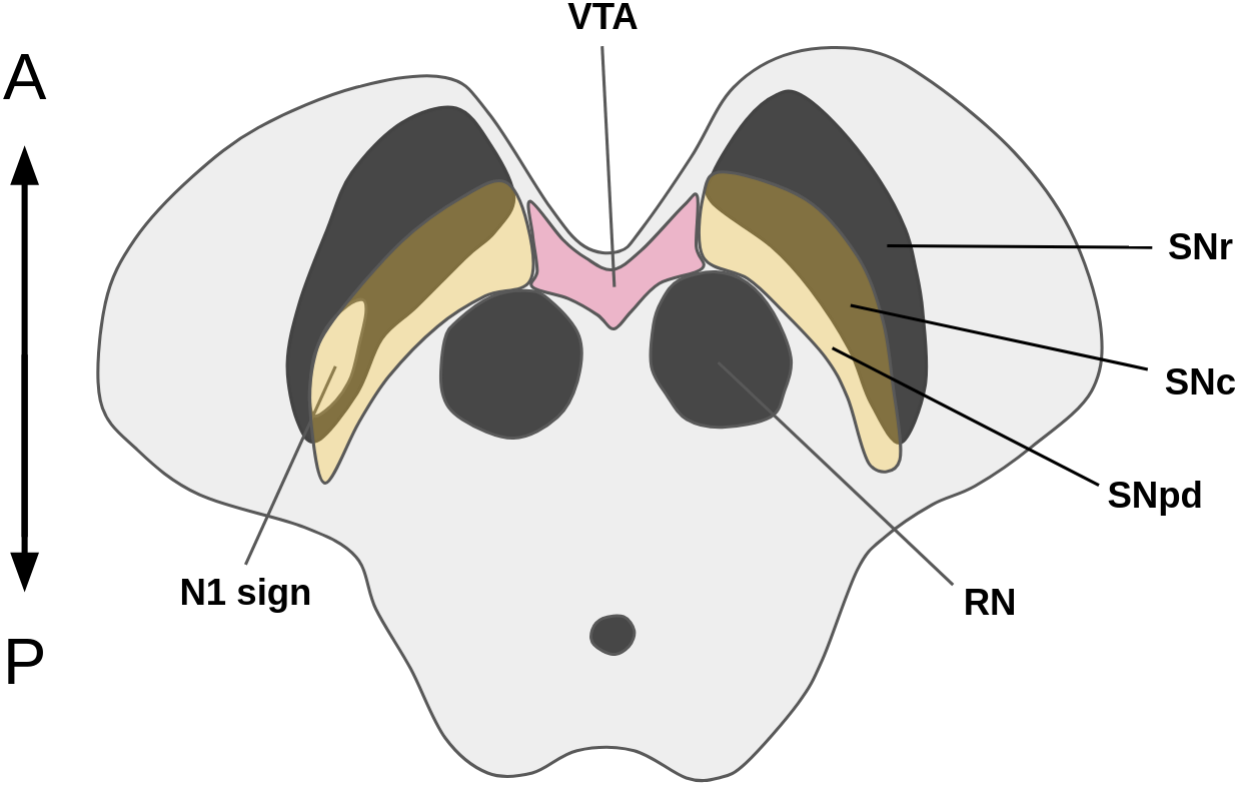
Schematic of the midbrain (axial slice) with MRI-relevant features highlighted. Both the SNr and SNc appear hypointense in susceptibility-weighted images, while neuromelanin in the SNc, SNpd, and VTA appear hyperintense in NM-MRI. The N1 sign is typically lost uni- or bilaterally in PD, which is understood to happen as neuromelanin degrades and releases its chelated iron, thereby increasing magnetic susceptibility. N1, nigrosome-1; VTA, ventral tegmental area; RN, red nucleus; SNc, substantia nigra *pars compacta*; SNr, substantia nigra *pars reticulata*; SNpd, substantia nigra *pars dorsalis*; A, anterior; P, posterior.

The anatomy of the SN is exceedingly intricate, which makes it challenging to discern its constituent parts in MRIs as well as in histological sections (Lehéricy et al., 2014; Massey et al., 2017). Principally, it comprises two main components: the *pars reticulata* (SNr) and the SNc. The SNc, which is the primary site of neurodegeneration in PD, sits dorsally adjacent to the larger SNr (Fig. 1).

Different MRI sequences are variably sensitive to signals from these midbrain substructures (Rua et al., 2021). High concentration of iron makes susceptibility-weighted imaging (SWI) excellent for imaging the SN, particularly the *pars reticulata*. However, due to age-related accumulation of iron in the *pars compacta*, it can be difficult to distinguish the SNr from the SNc from SWIs alone. Another method of increasing relevance to PD is NM-sensitive MRI (henceforth referred to simply as NM-MRI). NM is a byproduct of catecholamine metabolism, and is therefore specifically localized to the SNc and adjacent dopaminergic structures: the ventral tegmental area (VTA) and SN *pars dorsalis* (SNpd), while absent in the SNr. NM is understood to be neuroprotective both by effectively removing excess dopamine and by chelating iron and other metals, thereby preventing oxidative damage (Sulzer et al., 2018). Magnetization transfer (MT) between pools of free protons (water) and of restricted protons of the NM-metal complex can be exploited to achieve a magnetization transfer contrast (MTC) through the application of a MT pulse to indirectly visualize NM non-invasively (He et al., 2023). Neurodegeneration of the dopaminergic neurons of the SNc concurs with a depigmentation of NM, which, in turn, releases the previously chelated iron into the extracellular space, turning visible on iron-sensitive images like SWI and quantitative susceptibility maps (QSM). This is especially pronounced in nigrosome-1 (N1), located in the dorsolateral portion of the SNc, displayed as a loss of the N1-sign (He et al., 2023). Together, these sequences have shown much promise as biomarkers of PD (He et al., 2021, 2023; Sulzer et al., 2018). Consequently, the development and open sharing of automated tools for segmenting the structures is of great interest.

Atlas-based methods are traditionally used to segment the SN and are based on coregistering the image to a reference template for which the structure of interest is already labeled. This transformation is obtained by quantifying a goodness-of-fit between the transformed input image and the standard space image by optimizing a cost function like mutual information. This works well as a general purpose approach, and the same transformation can be reused for multiple structures. However, it is comparatively slow, requires additional storage space, and may be sub-optimal for small structures that have a proportionally small contribution to the total cost function. Another weakness to volumetric atlases may arise from a bias to the average population from which they were created, and perform systematically worse in less represented populations (Nakamura et al., 2018), which generalizes to neuroanatomical differences between groups of health and disease.

Some of these challenges have been addressed: the PD-25 subcortical atlas was constructed from a cohort of 25 PD patients to alleviate the issue arising from disease-specific pathology, as well as providing templates for multiple MR sequences (Xiao et al., 2015), although NM-MRI was not included. There is, nonetheless, a growing number of works on automating SN segmentation from NM-MRI, using either traditional methods (Ariz et al., 2019; Biondetti et al., 2020; Jin et al., 2022), or deep neural networks (Ariz et al., 2024; Gaurav et al., 2022; Krupička et al., 2019; Le Berre et al., 2019; Martínez et al., 2023). While these methods have proven helpful in deriving PD biomarkers from NM-MRI, it has made little impact on the space of publicly available models, with very few exceptions (Biondetti et al., 2020). This complicates the evaluation of model performance across cohorts, sites and pulse sequences, as well as necessitating labs to develop their own in-house methods.

In this work, we set out to train a neural network for segmenting the NM-hyperintensity of the SN, as an approximation of the SNc, with the purpose of expanding the currently small repertoire of public tools. We experimented with a wide array of MR contrasts (NM-MRI; true susceptibility-weighted imaging, tSWI; quantitative susceptibility mapping, QSM; diffusion tensor imaging, DTI; T1-weighted; T2-weighted; and fluid-attenuated inversion recovery, T2-FLAIR) from a mixture of PD patients and healthy controls, to evaluate segmentation performance in both single- and multiparametric models. We observed that models trained on NM-MRI from a single site would readily fail on out-of-site data, but we were able to improve the cross-site generalizability of the model by including multiparametric MRIs in the training set and applying aggressive data augmentation.

## 2 Material and Methods

### 2.1 Participants

A mixture of patients with PD and healthy control subjects were obtained from a Norwegian cohort, 77 of which were from Haukeland University Hospital, Bergen, and 5 subjects from St. Olavs hospital, Trondheim, and 27 subjects from the London Movement Disorders Centre, Ontario, Canada, all as part of the STRAT-PARK cohort (Stige et al., 2024). Disease status was confirmed by [^123^I]ioflupane SPECT as previously described (Tzoulis et al., 2013), and patients were rated using the Movement Disorder Society-sponsored revision of the Unified Parkinson’s Disease Rating Scale (MDS-UPDRS) scale (Goetz et al., 2008). Data acquisition was conducted in accordance with the ICH E6 Good Clinical Practice Guidelines and has been approved by the Regional Committee for Medical and Health Research Ethics, Western Norway (REK 74985) (Stige et al., 2024). All study participants provided written informed consent. Scans from an additional 209 subjects were downloaded from the Parkinson’s Progressive Markers Initiative (PPMI) database for validation purposes, comprising PD, prodromals, healthy controls, and SWEDD subjects (Scans Without Evidence of Dopaminergic Deficit). Demographics of the different cohorts are summarized in Tables 1, 2 and 3. The PPMI scans were split into two groups: one for segmentation validation (PPMI-GRE, N = 22) and one for clinical validation (PPMI-TSE, N = 187), and are therefore reported in separate tables.

**Table 1.** Participant summary of Norwegian cohort.

	PD	Control	P-value
N	58	22	
Male/female	37/21	13/9	0.797^a^
Age (years)*	71 (64,76)	68 (63,72)	0.147^b^
Disease duration (years)*	6 (4,8)	-	-
TD/PIGD/mixture	21/28/9	-	-
MDS-UPDRSIII*	25 (20,30)	-	-
Dominant side (left/right)	33/22	-	-

Data not shown for two participants who were later found to have atypical parkinsonism.

* Numbers are reported as median (25th percentile, 75th percentile).

The PD group and HC group were tested against the null hypothesis of an identical distribution with: ^a^Fisher’s exact test; ^b^Mann-Whitney U test.

TD, tremor dominant; PIGD, postural instability gait difficulty.

**Table 2.** Participant summary of the external cohorts (Canada and PPMI) for segmentation validation.

	Canada		PPMI	
	PD	Control	PD	Prodromal
N	25	2	6	16
Male/female	16/9	0/2	4/2	8/8
Age (years)*	62 (59,70)	66 (-)	62 (59,67)	70 (62,74)
Disease duration (years)*	6 (5,10)	-	-	-
TD/PIGD/mixture	9/10/0**	-	4/2/0	3/5/0***
MDS-UPDRSIII*	31 (24,35)	-	17 (11,23)	3 (2,6)

* Numbers are reported as median (25th percentile, 75th percentile).

** Numbers do not add up due to missing data.

*** Some participants had tremor and PIGD scores of zero, and were not classified into either motor subtype.

TD, tremor dominant; PIGD, postural instability gait difficulty.

**Table 3.** Participant summary of the clinical validation cohort (TSE-FS, PPMI), used for ROC-AUC analysis.

	PD	Control	SWEDD
N	124	31	32
Male/female	76/36	25/6	21/11
Age (years)*	65 (56,71)	64 (55,72)	61 (54,71)
TD/PIGD/mixture	66/37/5**	-	16/11/3**
MDS-UPDRSIII*	21 (13,28)	-	11 (7,15)

* Numbers are reported as median (25th percentile, 75th percentile).

** Some participants had tremor and PIGD scores of zero, and were not classified into either motor subtype.

TD, tremor dominant; PIGD, postural instability gait difficulty.

### 2.2 Imaging protocol

#### 2.2.1 Norway

Participants were imaged using a 3T MRI scanner (Biograph mMR, Siemens Healthineers, Erlangen, Germany) with a 12-channel head coil and 4-channel neck coil, both receive-only. The sequences and selected parameters were as follows: 1.0 mm^3^ isotropic 3D sagittal MPRAGE, repetition time (TR) = 2400 ms, echo time (TE) = 2.26 ms, inversion time (TI) = 900 ms, flip angle (FA) = 8°, bandwidth = 200 Hz/Px, matrix = 256 × 256, 192 slices, acquisition time (TA) = 5:35; 2D transverse T2-weighted turbo-spin echo, TR/TE = 3000/100 ms, FA = 150°, bandwidth = 220 Hz/Px, matrix = 512 × 384 and 27 slices with voxel size 0.43 × 0.57 × 4.0 mm^3^, TA = 2:26; 2D transverse T2 FLAIR, TR/TE = 11360/124 ms, TI = 2700 ms, FA = 150°, bandwidth = 260 Hz/Px, matrix = 384 × 256, voxel size = 0.67 × 1.0 × 2.7 mm^3^ with 80 slices, and TA = 3:03; 2.0 × 2.0 × 2.0 mm^3^ 2D transverse echo-planar diffusion-weighted images (DWI) with 30 + 30 directions over 2 b-value shells (1000 and 2500 s/mm²), phase encoding direction: anterior-to-posterior and one in posterior-anterior for distortion correction (a minority were obtained with right-to-left phase encoding instead of posterior-to-anterior), TR/TE = 5900/113 ms, FA = 90°, bandwidth = 1518 Hz/Px, 122 × 122 matrix and 25 slices, TA = 6:19; susceptibility-weighted images were obtained with a 3D transverse multi-echo gradient echo (GRE) sequence (described in Chen et al., 2018), to yield both T1-weighted (FA = 6°, TA = 4:57) and proton-density weighted (FA = 27°, TA = 4:57) images, of resolution 0.67 × 1.0 × 1.34 mm^3^ and matrix 384 × 256, 112 slices, TR = 29 ms, TEs = 7.5, 15.0, 22.5 ms, bandwidth = 210, 160, 160 Hz/Px; NM-sensitive contrast was achieved with a 3D transverse gradient echo magnetization transfer contrast (MTC) on-resonance of 0.67 × 1.0 × 1.34 mm^3^ voxels, matrix = 348 × 256, 48 slices, TR = 62 ms, TEs = 7.5, 15, 22.5, 30, 37.5 ms, bandwidth = 180 Hz/Px, FA = 12°, and 30°, TA = 4:38 (per flip angle).

#### 2.2.2 Canada

Twenty-seven subjects were imaged on a 3T MRI scanner (Discovery MR750W, GE Healthcare, Milwaukee, WI) with a 32-channel phased array head coil using two 3D transverse multi-echo GRE sequences, both with a MTC pulse. The imaging parameters for these sequences were: TR = 72 ms, TEs = 7.5, 15, 22.5, 30 ms, flip angle for the first sequence = 12°(TA = 5:56) and for the second sequence = 30°(TA = 6:04), bandwidth = 139 Hz/Px, matrix size = 384 × 256, voxel resolution = 0.67 × 1.0 × 1.34 mm^3^. 3D transverse T1-weighted IR-FSPGR scans were also acquired, with TR/TE = 9.008/3.892 ms, TI = 400 ms, FA = 11°, bandwidth = 177 Hz/Px, TA = 3:18, and a resolution of 0.88 × 0.88 × 1.0 mm^3^.

#### 2.2.3 PPMI

Scans from 209 subjects derived from a variation of sites and scanners were downloaded from the PPMI database. This included 22 anisotropic 2D GRE-MT scans and 187 dual-echo TSE scans with a fat saturation (FS) pulse. The TSE-FS scans were acquired on 3T Siemens scanners, with a resolution of 0.9 × 0.9 × 3 mm^3^. The majority GRE-MT scans had a resolution of 0.5 × 0.5 × 1.5 mm^3^ (6 subjects deviated with a slice thickness of 2.0 mm). For the 22 subjects with GRE-MT scans, we also downloaded high-resolution isotropic 1.0 mm^3^ T1-weighted anatomical scans from 12 of those and T2-FLAIR scans from the remaining 10 of the 22 subjects. Because of their diverse origins, a minority of the scans deviated slightly from the standard image resolution. For a complete description of the imaging protocol, visit www.ppmi-info.org.

#### 2.2.4 DaT

All patients of the Norwegian cohort underwent SPECT 3 hours after intravenous injection of 185 MBq [^123^I]ioflupane (DATScan®) according to our previously published protocol (Tzoulis et al., 2013). Images were acquired on a 2-headed SPECT camera (Siemens Symbia Intevo Bold; Siemens Healthineers, Hoffman Estates, IL) using low-energy high-resolution collimators. For quantification, images were processed on a Xeleris 4 workstation (GE Healthcare). Images were reconstructed by filtered back projection using a 10th-order Butterworth filter with a critical frequency of 0.5 and Chang’s attenuation correction and thereafter processed with the fully automatic quantification software ‘DATQuant’ which calculates bilateral specific binding ratios in the caudate nucleus, and anterior and posterior putamen (Tzoulis et al., 2013). Parkinson’s disease was confirmed if the specific binding ratio (SBR) in the posterior or entire putamen was lower than 2 standard deviations below the mean in the software’s age-specific normal database. For evaluating the ability to distinguish left-dominant from right-dominant patients, we computed the ratio of the SBR in the left hemisphere to the right hemisphere, and calculated the common language effect size, defined as the the proportion of cases where the left/right ratio was favorable out of all possible pairs of samples across the left-dominant and right-dominant groups. (McGraw & Wong, 1992).

### 2.3 Image preprocessing

Using the FMRIB Software Library (FSL; version 6.0.5) (Jenkinson et al., 2012), DWIs were first corrected for motion and distortion, before running eddy-current correction. A diffusion tensor model was fitted onto the preprocessed data with the DiPy library (version 1.5.0) (Garyfallidis et al., 2014). From this, we calculated maps of fractional anisotropy (FA), mean, axial and radial diffusivity (MD, AD and RD, respectively). Susceptibility-weighted images were processed using STAGE (version 2.8.1) software (SpinTech, Inc., Bingham Farms, MI, United States) (Gharabaghi et al., 2020) to obtain true SWIs (tSWI, referred to as simply SWI in the figures below) and quantitative susceptibility maps (QSMs) using the sophisticated harmonic artifact reduction approach (kernel = 6) for background field removal, and a geometry constrained iterative k-space algorithm was used to apply the inverse filter (regularization threshold = 0.1, iterations = 4) to obtain the susceptibility maps (Schweser et al., 2011; Tang et al., 2013). All contrasts were affinely coregistered and resampled to their respective NM image (within-subject) with FSL’s FLIRT (Jenkinson et al., 2012) using mutual information with 6 degrees of freedom, prior to training the deep learning models. We chose the NM image as a reference because the SN was labeled from these images, thus avoiding a resampling of the masks. We reused the affine matrix obtained from the MD maps to transform the remaining DTI maps, as they are aligned by construction. PPMI scans from the same subject and session were coregistered rigidly with FLIRT and averaged to produce the final images.

### 2.4 Experimental overview

**Fig. 2.**
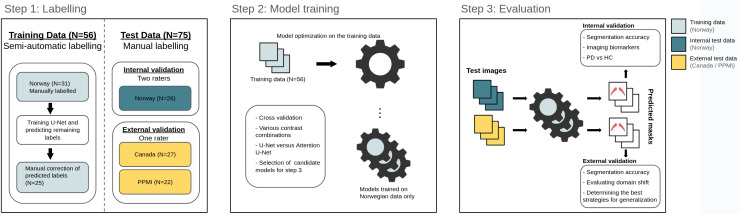
Experimental pipeline.

Following a canonical design, the Norwegian participants were split into a training (N = 56) and a test (N = 26) set, for developing and evaluating the neural network models, and deriving imaging biomarkers (Fig. 2). In addition, two external cohorts (Canada, N = 27 and PPMI, N = 22) were included as a validation to evaluate segmentation performance on out-of-distribution populations, in contrast to the in-distribution test set of the Norwegian cohort, which was derived from the same population as the training set. The test scans were labeled independently and blinded to disease status by a radiologist of 6 years experience (T.N.) and a non-clinician (P.L.), to assess inter-rater variability, but only the labels from T.N. were used in the testing phase. The scans from the external validation cohorts were labeled by P.L. only. For the training set, 56 Norwegian subjects were labeled in part manually (P.L.) and partly automatically: 31 of the NM scans were segmented and used to train a U-Net, from which we predicted the remaining 25 unlabeled NM scans of the training set. Manual corrections were made to the predictions before they were pooled together with the original 31 subjects, constituting the complete training set.

Once the NM scans from all participants had been segmented, we used 6-fold cross validation (CV) on the training set to determine the optimal neural network model, data input size, and MRI contrast combinations (described in detail in Section 2.7). Due to the utilization of multiple MRI contrasts in some models, the data were always split on the level of the subject, to prevent different contrasts from the same individual to end up in separate sets. Once candidate models had been selected from CV, they were retrained on the whole training set. Finally, the models were evaluated on the test set, from which we derived imaging biomarkers for separating PD patients from controls. The two external validation sets were used to determine cross-site generalizability of the trained models, and compare strategies for managing data drift. In the end, after the methods had been validated, we trained a model on the three above mentioned cohorts, which was evaluated for clinical performance on the 187 TSE-FS scans from PPMI.

### 2.5 Manual labeling

The region of interest (ROI) in the NM images was delineated manually using ITK-SNAP (version 3.8.0) (http://www.itksnap.org) (Yushkevich et al., 2006). For the multi-echo scans, this was performed on the first echo (7.5 ms) of the 12° flip angle image. First, the image contrast was set using the “Auto Adjust Contrast” option in ITK-SNAP. The midbrain was localized in the axial plane, and the perimeter of the SN hyperintensity was traced and filled in with the smooth curve option in “Polygon Mode”, slice by slice in 5–8 contiguous axial slices, the exact number depending on the apparent brightness relative to the surrounding tissue. Refinements were made in “Paintbrush Mode” with a brush size of 1 voxel. The zoom factor was actively varied in order to capture both the overall shape as well as finer details.

The inter-rater agreement between volumes X and Y was determined by the Dice-Sørensen similarity coefficient (DSC):

$$DSC=\frac{2\left|X\cap Y\right|}{\left|X\right|+\left|Y\right|},$$

where |⋅| denotes the volume, and ∩ the intersection.

### 2.6 Atlas labeling

As a baseline comparison to our method, we performed atlas-labeling of the SNc with a minimal pipeline. First, each individual NM image was rigidly aligned to their respective T1-weighted anatomical scan using a symmetrical block-matching algorithm and normalized cross correlation (Modat et al., 2014). The T1-weighted images were, in turn, non-linearly transformed to a 1 mm^3^ isotropic T1-weighted template (Biondetti et al., 2020, 2021) (available from https://github.com/emmabiondetti/substantia-nigra-neuromelanin), with cubic B-splines (Free-Form Deformation) and trilinear interpolation by optimizing the normalized mutual information and bending energy (Modat et al., 2010). These two transformations were sequentially applied to acquire the NM images in the template space. The NM images of the Norwegian cohort were averaged to produce a mean NM template. By manually labeling the hyperintensity in this template, we constructed a symmetric SNc atlas (referred to as atlas_pl). We also made comparisons with the atlas provided from the aforementioned GitHub repository by merging the three subsegments into a single ROI (referred to below as atlas_eb). Next, the compound transformation was reversed in order to obtain the mapping from template space to native NM space. These were applied to the SN atlas, and to the crus cerebri, as a reference for calculating NM contrast, isolated from the background region provided in the same repository, yielding the final masks. All registrations were achieved with NiftyReg (version 1.5.76) (Modat et al., 2010), and T1-weighted images had been skull-stripped prior to calculating the transformations, using FSL’s Brain Extraction Tool (Jenkinson et al., 2012). Because we did not have access to T1w scans for 10 of the 22 PPMI subjects, we synthesized 1 mm^3^ isotropic MPRAGE contrasts from their respective T2-FLAIR scans (Iglesias et al., 2021, 2023) prior to performing the steps described above.

### 2.7 Deep learning

To build and train deep neural networks for segmentation, we made use of the U-Net architecture (Ronneberger et al., 2015) and Attention U-Net (Oktay et al., 2018), implemented in FastMONAI (version 0.3.9) (Kaliyugarasan & Lundervold, 2023), built on the Pytorch-based MONAI (version 1.2.0) (Cardoso et al., 2022; Paszke et al., 2019) and FastAI (version 2.7.12) (Howard & Gugger, 2020) libraries. To handle the imminent class imbalance in the images (i.e., the ROI is small and far outnumbered by background voxels in the mask), we used the focal Tversky loss function, which generalizes the Dice loss (Abraham & Khan, 2019). The training process was optimized using the Ranger optimizer (Wright & Demeure, 2021), and the learning rate was scheduled with flat and cosine annealing. We added data augmentation during training, starting out with mild affine transformations well within anatomical variability: displacement, rotation, scaling, as well as mirroring the left-right axis. We later amplified and expanded the list with motion artifacts and bias field simulations, elastic transformations, random noise, and gamma augmentation. Technical specifications are provided in the Supplementary Material. Models were trained in a number of different combinations to explore performance as a function of the input MR contrast(s). We tested each contrast (T1, T2, FLAIR, tSWI, QSM, NM, FA, MD, AD, RD) separately, as well as in multi-channel networks for each possible pair, to study potential synergistic effects. Finally, we constructed one model (referred to as “all-in-one”), which was trained on images from every available contrast, but as a single-channel architecture, meaning that each contrast from the same participant was fed into the model as a separate sample. To avoid the possibility of data leakage, we separated our training and test data based on participant ID. Effects from multi-contrast models were explored with CV using the training set only, before validating them on the test set. All models were trained exclusively on the Norwegian cohort and validated on the Canadian and PPMI cohorts to address the generalization problem and to provide a realistic demonstration of how models behave in exposure to truly independent test sets.

The final models were evaluated both with the DSC and surface-based metrics: the mean surface distance (MSD) and the 95th percentile Hausdorff distance (HD95). By letting the surface distance from a point a on the predicted surface A to the ground-truth surface B be defined as the minimum of the Euclidean distances, $∥\cdot {∥}_{2}$, between a and all points b∈B :

$$d\left(a,B\right)=\underset{b\in B}{\text{min}}∥a-b{∥}_{2},$$

then the MSD is the average of the distance vectors d(A,B) and d(B,A). HD95 is obtained by first taking the 95th percentile of the one-sided distance vectors:

$$h\left(A,B\right)=P_{95}\left(d\left(A,B\right)\right),$$

and then selecting their maximum:

HD95=max(h(A,B), h(B,A))

Both surface metrics are reported in millimeters.

### 2.8 Imaging biomarkers

NM-content in the SN was quantified as the contrast-to-noise ratio (CNR) relative to a reference region in the crus cerebri: $(I_{sn}-I_{cc})/SD_{cc}$ , where I is the mean intensity and SD is the standard deviation. The related expression contrast ratio (CR) was defined as $(I_{sn}-I_{cc})\text{​}/\text{​}I_{cc}$ . NM volume was normalized by total brain volume, which we estimated from T1-weighted images using FastSurfer (Henschel et al., 2020).

## 3 Results

This section presents a comprehensive analysis of our deep learning-based approach to segment the substantia nigra in Parkinson’s disease (PD) using multiparametric MRI. We begin by examining inter-rater variability in manual segmentation, which serves as our ground truth. We then detail our combinatorial screening process to identify optimal MRI contrast combinations. Next, we describe the optimization and testing of our models, including a comparison with atlas-based segmentation methods. We evaluate our models’ ability to generalize by testing on out-of-site data and explore the effects of aggressive data augmentation. Finally, we validate our approach clinically by analyzing SN volume and neuromelanin contrast-to-noise ratio, assessing discrimination performance between PD patients and healthy controls, and investigating the relationship between SN metrics and symptomatic laterality in PD. Throughout this section, we compare the performance of our deep learning models with traditional atlas-based methods and manual segmentation, providing a comprehensive evaluation of our approach’s strengths and limitations.

### 3.1 Segmentation

#### 3.1.1 Inter-rater variability

**Fig. 3.**
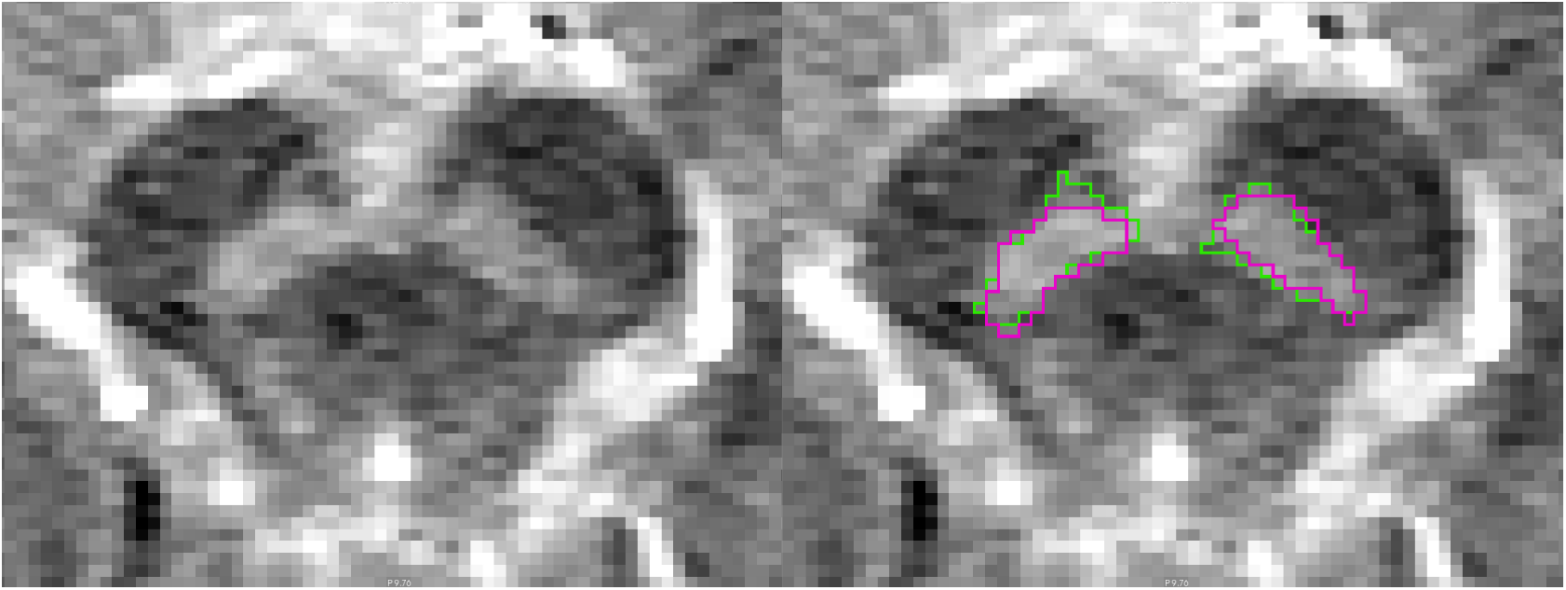
NM-MRI of the midbrain (axial slice) with outlined masks of the substantia nigra hyperintensity from the two raters on the right.

The 26 images comprising the test set from the Norwegian cohort were labeled by both raters (Fig. 3). The inter-rater agreement between the two raters averaged at .698 , with a standard deviation of .108 .

#### 3.1.2 Combinatorial screening

**Fig. 4.**
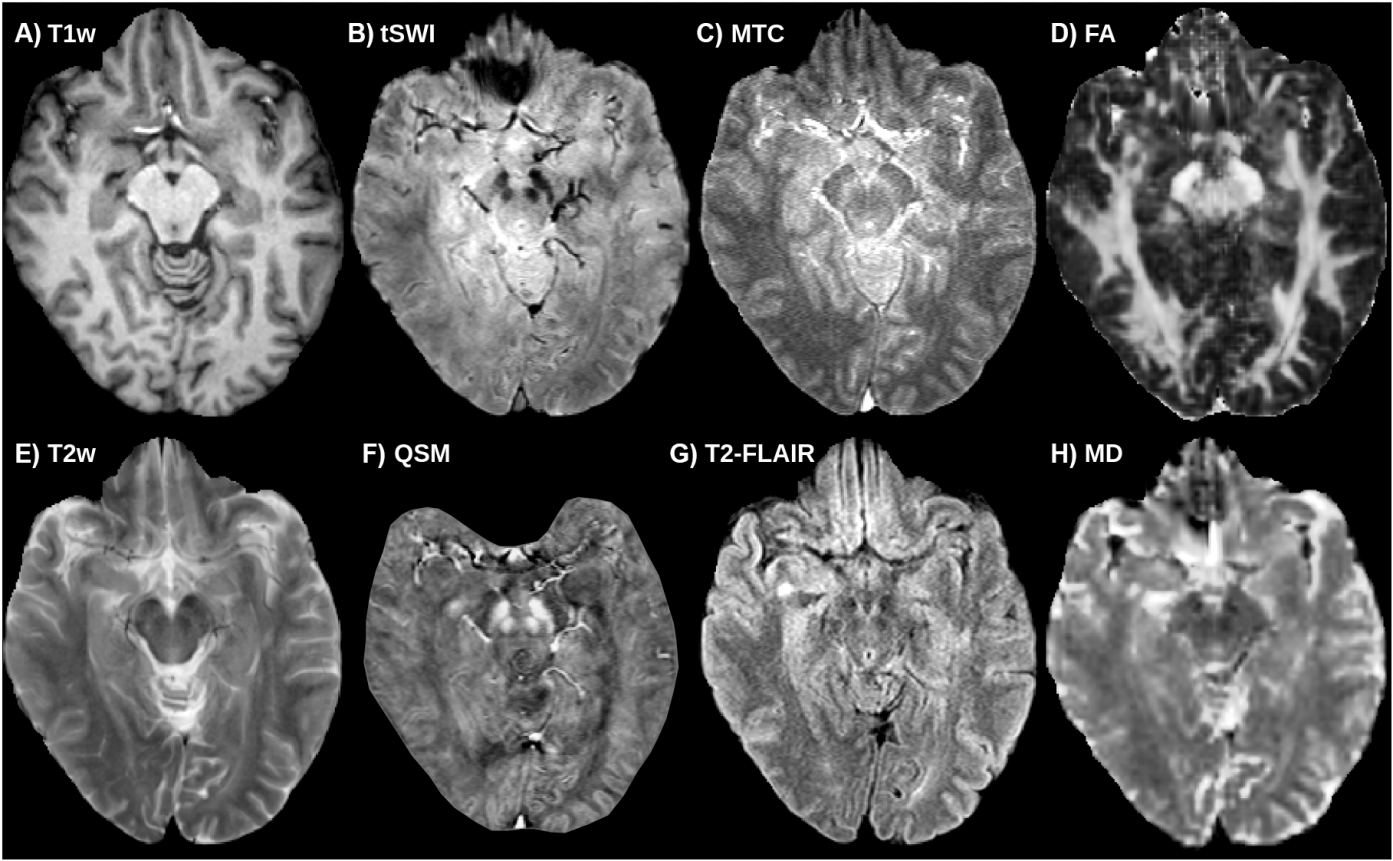
Coregistered multiparametric MRIs from a healthy control. Top row, from left: (A) T1w, (B) tSWI, (C) GRE-MTC (NM), (D) FA. Bottom row, from left: (E) T2w, (F) QSM, (G) T2-FLAIR, (H) MD.

**Fig. 5.**
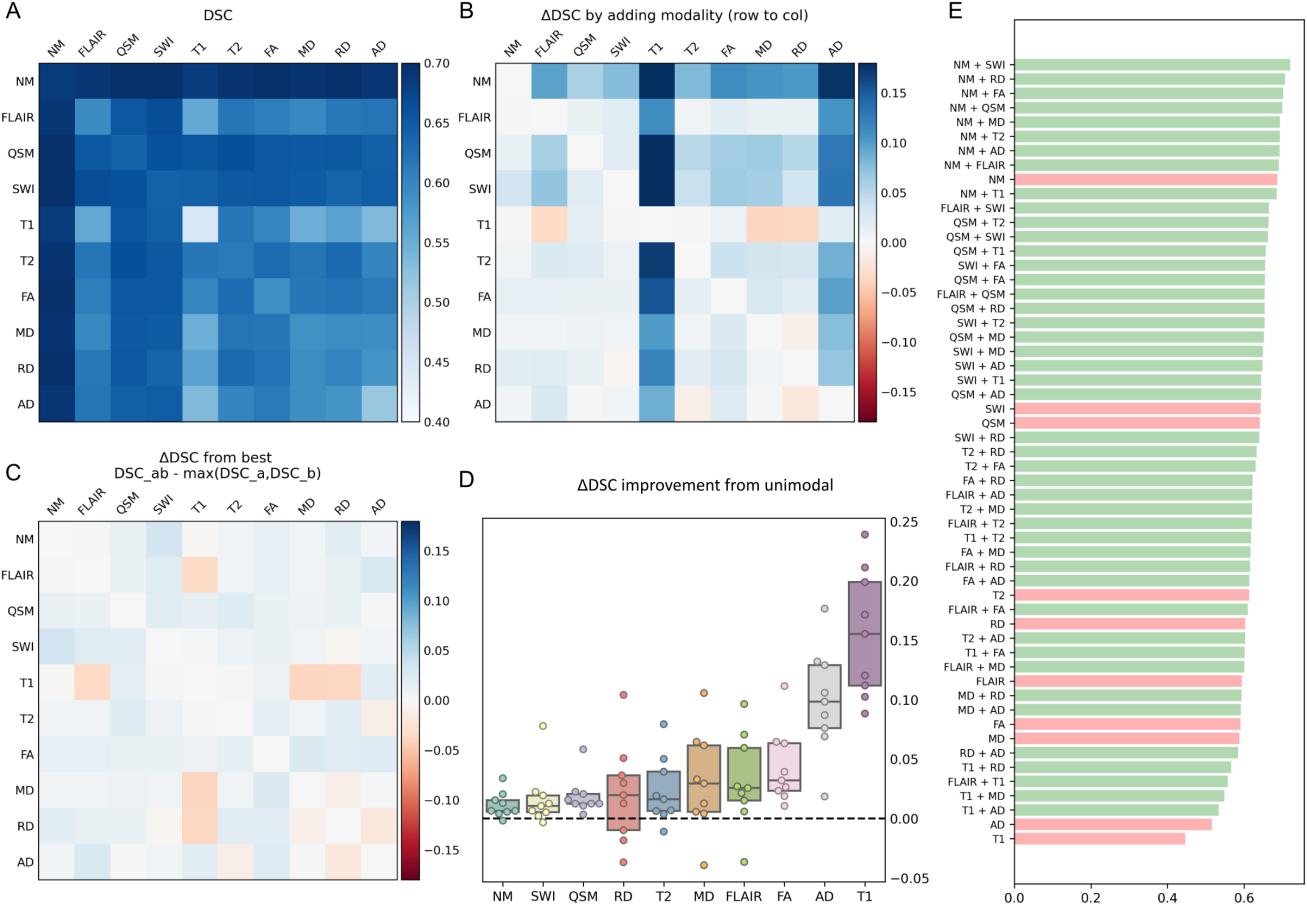
Comparative Dice scores of U-Net with multiparametric MRI. (A) DSC from all pairs, single-contrast scores on the diagonal; (B) ΔDSC by adding sequence at row i to that at column j: $e_{i,j}=DSC_{i,j}-DSC_{j,j}$ ; (C) ΔDSC of each pair versus the best of its constituents; (D) The “improvement potential” from baseline single-contrast model (corresponding to the columns in B); (E) Raw DSC sorted from highest to lowest (single-contrast models in red, pairs in green).

Prior to optimizing the model and training parameters, we wanted to identify the best performing combinations of contrasts (a subset of which shown in Fig. 4) from the training data. For the full spectrum of contrasts, see Supplementary Figure S2. We trained one U-Net for each of the 10 contrasts individually as well as for each of $\left(\begin{array}{c} 10\\ 2 \end{array}\right)$ = 45 possible pairs, to explore whether additional sequences in combination could be useful to bootstrap the training process. The procedure was repeated in three folds (46 training / 7 validation), from which we report the mean (Fig. 5).

The best performing model was the union of NM and tSWI, and the worst was singular T1, by a large margin. Although diffusion measures performed poorly in isolation, FA and RD ranked third and second when combined with NM (Fig. 5E).

**Fig. 6.**
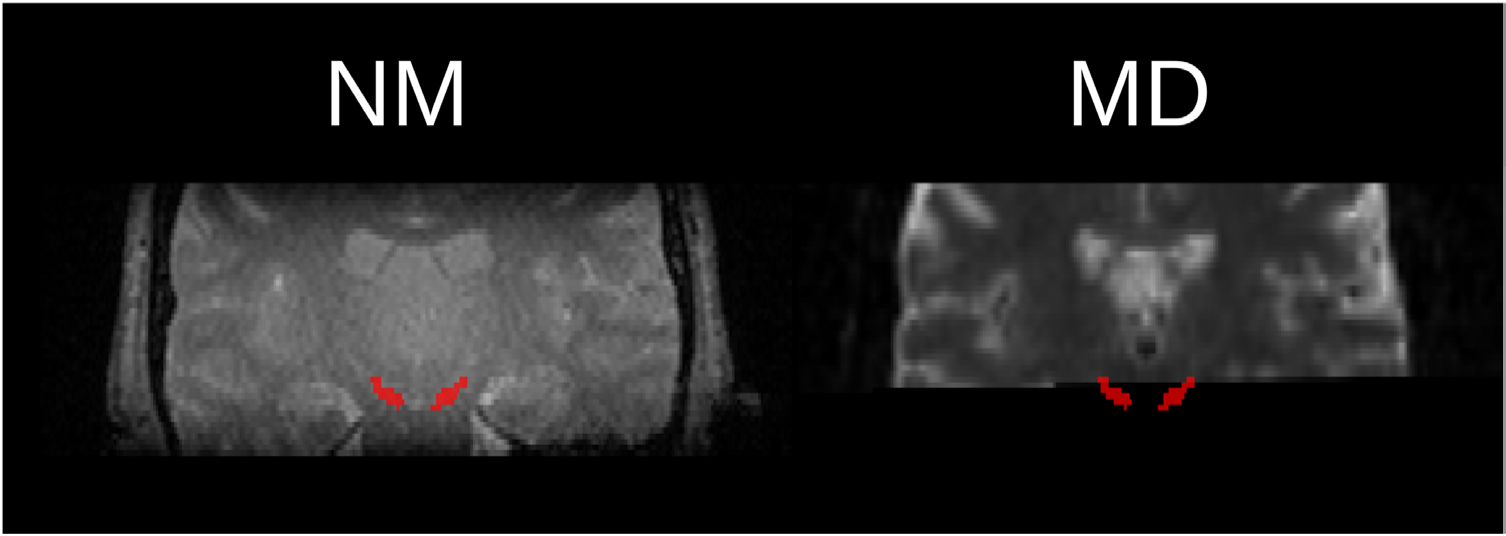
Extreme case showing NM-MRI (left) and MD map (mean diffusivity, right) from the same participant and slice, demonstrating a poor coverage of the SN (red) in the MD.

We constructed a matrix from all the scores, such that the entries $A_{i,j}$ represented the DSC from a model trained on contrast i and j, with single-contrast models on the diagonal. To elucidate how each union model performed relative to models trained on its constituent contrasts alone, we subtracted the values at the diagonal from their respective columns (Fig. 5B, D): $B_{i,j}=A_{i,j}-A_{j,j}$ . Thus, each row $B_{i}$ represents the influence of adding contrast i to models trained on the other contrasts, and the columns $B_{j}$ represent the improvement (or deterioration) a model trained on contrast j experiences from including additional contrasts. We observed that adding any arbitrary contrast to any other tended to improve the DSC, with the exception of T1, which exerted the opposite trend. When comparing the bi-contrast models with the best of its respective single-contrast models, we saw a weaker but still positive trend of improvement (Fig. 5C). DTIs exhibited the overall worst performance, but it is worth considering that a low field of view caused many to lose coverage of the SN, as illustrated in Figure 6.

#### 3.1.3 Model optimization and testing

A subset of the combinations (see Table 4) was selected for further optimization through a 6-fold CV on the training set. The selection was based on three considerations: inclusion of top-performing models, incorporation of notable combinations that outperformed their individual contrasts, and representation of single-contrast models for reference. Testing both U-Net and Attention U-Net, we explored performance with two image sizes by cropping: 144×144×48 and 96×96×48 . Due to high memory requirements with Attention U-Net, we could only train it on the smaller matrix size. We observed a consistent improvement in performance of Attention U-Net compared to U-Net after cross validation (Supplementary Fig. S1). Therefore, we decided to only take Attention U-Net to the final stage, where all models were retrained on the full training set. Because we wanted to exploit all the training data for the final models, leaving none for validation, the number of epochs was fixed to 150. Each model was evaluated on the test set, on the same set of contrasts it had been trained on. Out of our selected models, the NM-only model performed best on the test set, with a mean DSC of .749 on the test set (PD/HC = 0.71/0.78), doing marginally better than NM in conjunction with either tSWI, RD or FA (see Table 4). Unexpectedly, the previously observed performance boost from using multiple contrasts was not evident with Attention U-Net, despite being consistently observed in the screening with U-Net.

**Table 4.** DSC on the test set (N = 26) from a selection of models.

Model	Mean DSC
NM_AttentionUnet	0.749
all-in-one_AttentionUnet	0.746^a^
NM-RD_AttentionUnet	0.740
NM-FA_AttentionUnet	0.739
NM-tSWI_AttentionUnet	0.739
tSWI-FLAIR_AttentionUnet	0.683
tSWI_AttentionUnet	0.665
QSM_AttentionUnet	0.662
FLAIR_AttentionUnet	0.659
T2_AttentionUnet	0.650
RD_AttentionUnet	0.628
RD-FA_AttentionUnet	0.598
T1_AttentionUnet	0.396

a Evaluated on NM images.

One last model (“all-in-one”) was trained on all the available contrasts except AD and MD. Due to an increased sample size, the all-in-one model was trained for only 50 epochs. Here, we report its performance on NM images. For its performance on other contrasts, see Table S1 in the Supplementary Material. On the test set it reached a DSC of 0.746 (PD/HC = 0.72/0.77), essentially matching the best performing NM-only model. Moreover, when comparing the all-in-one model to the next-best single-sequence models like the tSWI, QSM, FLAIR and T2 models, it performed equally well or slightly better, indicating a good generalizability across sequences (Supplementary Table S1). Interestingly, if we trained the NM-only model on atlas_eb labels instead of manual masks, we could observe a moderate boost in DSC from 0.75 to 0.82, but we did not see the same effect on the all-in-one model.

#### 3.1.4 Comparison with atlas segmentation

We calculated the Dice coefficient separately for the test set and training set of the Norwegian cohort in order to ease comparisons with the deep learning models. The DSC was notably higher in the test set (0.63, atlas_pl; 0.51, atlas_eb) than in the training set (0.50, atlas_pl; 0.44, atlas_eb), reflecting a higher proportion of healthy controls in the test set (58%) than in the training set (13%). Note that the terms “training set” and “test set” only apply to the deep learning models. The atlases had variable performances across the different cohorts (Table 5) with atlas_eb performing better on PPMI images and atlas_pl better on the Canadian images. Atlas_pl was drawn on an averaged template derived from the complete Norwegian dataset, which is mirrored by a higher DSC score from atlas_pl on these images.

**Table 5.** DSC on all cohorts from atlas labeling.

Cohort	N	atlas_eb	atlas_pl
Norway (test)	26	0.507	0.629
Norway (training)	56	0.441	0.501
Canada	27	0.533	**0.583**
PPMI	22	**0.489**	0.462

The highest DSC is indicated in bold for the independent validation sets. The Norwegian data is not indicated because atlas_pl was created from the Norwegian data.

#### 3.1.5 The “all-in-one” model: prediction on out-of-site data

**Fig. 7.**
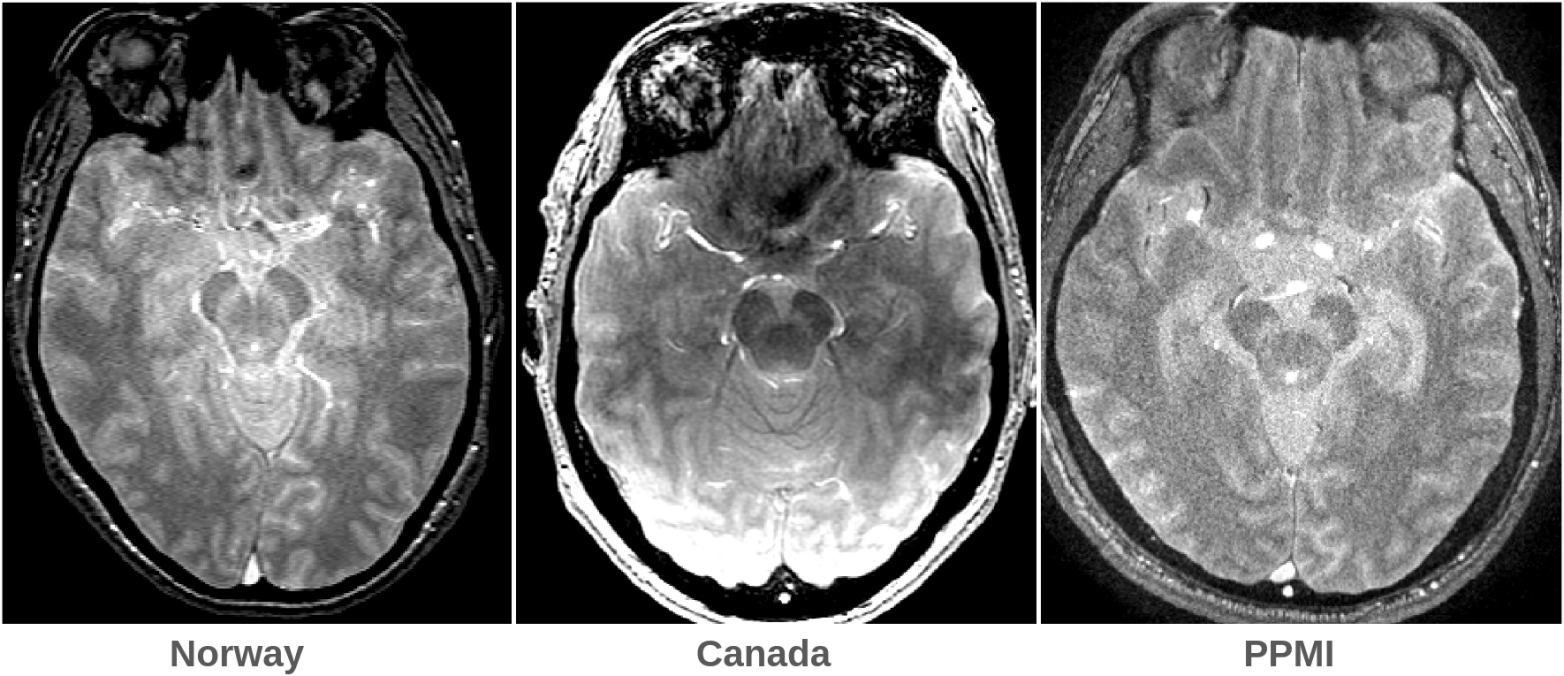
Representative NM-MRIs (axial view) from the three cohorts. Notice the inhomogeneity artifact in the center image.

The 27 NM-scans from the Canadian cohort and 22 from PPMI served as separate validation sets to test the models’ ability to generalize to out-of-site data. Despite using an essentially identical protocol, the Canadian MRIs were acquired on a different scanner (GE DISCOVERY MR750) with different interpolated spatial resolution and different MT pulse implementation, yielding different visual characteristics (Fig. 7), most notably stronger inhomogeneity. The PPMI scans looked qualitatively more similar to the Norwegian scans, despite a truncated field of view along the Z direction, and in many cases a lower SNR.

**Fig. 8.**
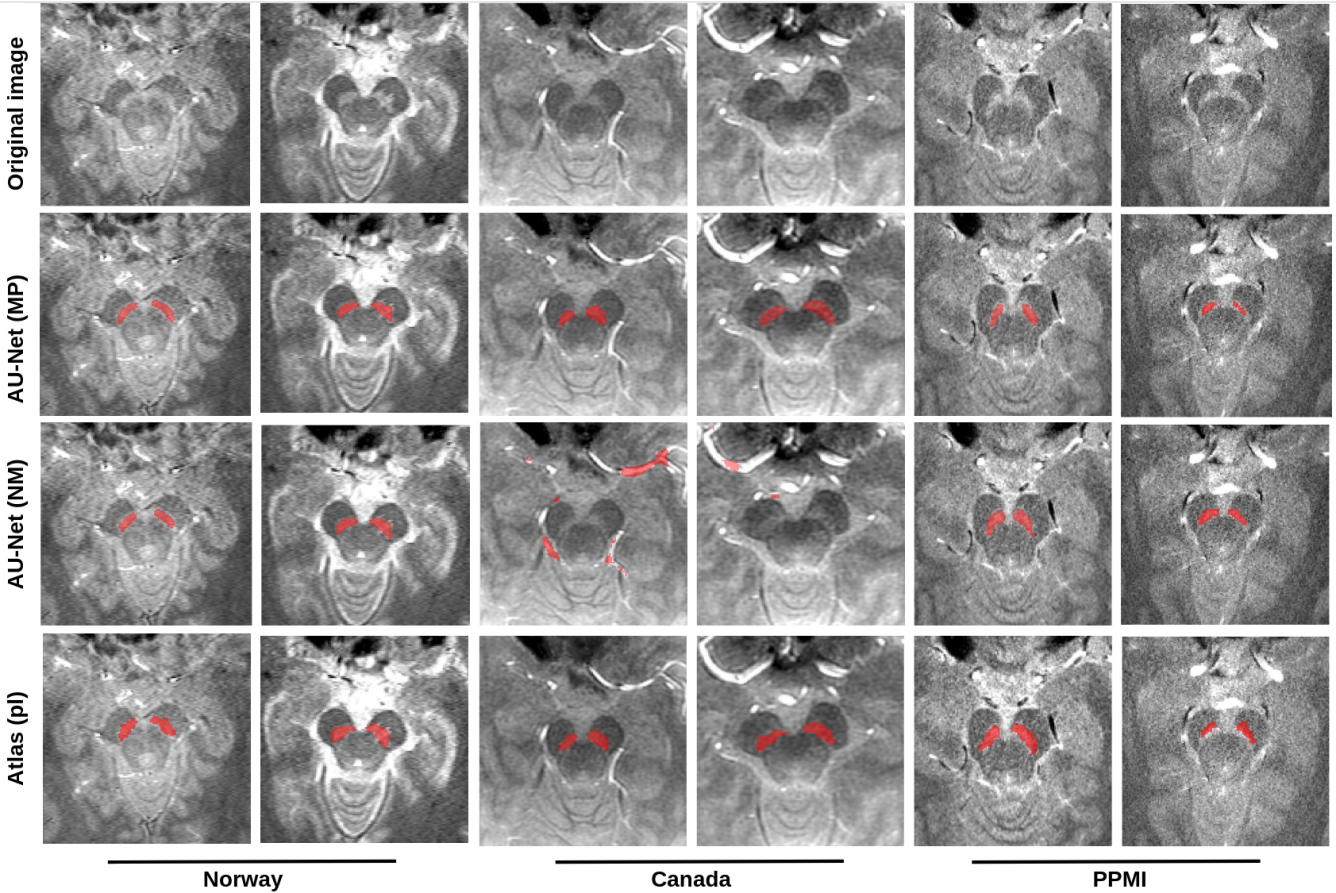
Model performance across sites (low augmentation). Both neural network models performed similarly on internal test data (Norway, first and second column), but the canonical NM-only model was unable to make a reasonable prediction on the Canadian cohort (3rd and 4th columns). On the PPMI cohort (5th and 6th columns), the NM-only model performed better than the multiparametric model. Our atlas at the bottom row for comparison. AU-Net (MP), Attention U-Net trained on multiparametric data (“all-in-one”); AU-Net (NM), Attention U-Net trained on NM-MRI only; and Atlas (pl), our atlas.

Although both the NM-only and “all-in-one” models had a similar performance on images from the Norwegian cohort (Table 4), the NM-model failed completely to even identify the SN in the majority of the Canadian MRIs, with a mean DSC of 0.007, instead mislabeling small high-intensity voxel clusters (Fig. 8). The “all-in-one” model also dropped in performance compared to how it fared on the Norwegian test set, but the regularization effect from the multiparametric strategy was helping, landing at an average DSC of 0.44. This effect was more questionable in the PPMI cohort, in which the NM-only model (DSC = 0.36) outperformed the “all-in-one” model (DSC = 0.22), although both did worse than simple atlas labeling. When we repeated the experiment with U-Net as opposed to Attention U-Net, (i.e., with one NM-model and one multicontrast model), the multiparametric model did slightly better (DSC = 0.40) than the NM model (DSC = 0.32) on PPMI. On the Canadian validation set, we saw a similar pattern to the case with Attention U-Net, where the model could be improved by training on multiparametric MRI (from 0.015 to 0.41).

#### 3.1.6 Aggressive augmentation

The multiparametric approach appeared to yield a net benefit over the canonical model on external datasets, but not to a degree that would be sufficient for real-world application. After noting that the external data suffered from strong bias fields, we decided to expand the augmentations during training with simulations of MR artifacts, including adding a bias field and motion artifacts, as well as gamma augmentation, elastic deformations and random noise (Pérez-García et al., 2021). The previous augmentations were kept, but we enhanced the amount of rotation, translation and scaling. We applied the changes both to the NM model and the multiparametric model and recorded the results. Again, the DSC on the Norwegian test set was largely unaffected, but the performance on the external validation sets was overall improved (Table 6), reaching a DSC of 0.68 and 0.66 on the Canadian and PPMI cohorts respectively. The difference between the canonical approach and the multiparametric approach was now less pronounced, but leaning in a slight favor of the “all-in-one” model. The MSD and HD95 also improved with the multiparametric strategy (Table 7), supporting the notion of a small benefit to training on multiparametric MRI. Note that all the models were only trained on the Norwegian cohort.

**Table 6.** DSC after aggressive augmentation.

Cohort	N	NM-only	all-in-one
Norway test set (in-distribution)	26	**0.745**	0.742
Canada (out-of-distribution)	27	0.671	**0.679**
PPMI (out-of-distribution)	22	0.651	**0.660**

The highest DSC is indicated in bold.

**Table 7.** HD95 and MSD after aggressive augmentation.

		HD95 [mm]		MSD [mm]	
Cohort	N	NM-only	all-in-one	NM-only	all-in-one
Norway test set (in-distribution)	26	1.68	**1.57**	0.39	**0.38**
Canada (out-of-distribution)	27	7.66	**3.68**	1.23	**0.95**
PPMI (out-of-distribution)	22	2.63	**2.27**	0.56	**0.52**

The smallest surface distances are indicated in bold.

### 3.2 Clinical validation

The following analyses were performed exclusively on the Norwegian cohort and TSE scans from PPMI, because the remaining cohorts were highly imbalanced in terms of disease status (all but two subjects from Canada had PD, and the majority of the GRE scans from PPMI were prodromal)—such that any attempt to adjust for the site would have complicated the analysis and biased the results.

#### 3.2.1 Volume and NM contrast-to-noise ratio

With the corrected volumetric measurements, the manual labels yielded the largest group difference (p = 0.003, Mann-Whitney U-test) between PDs and controls for the whole dataset. Despite a similar trend in the test set, the effect was small (p = 0.15). The two DL models replicated this somewhat in the whole dataset (p = 0.021, p = 0.045), but this may be explained by overfitting. In the test set, only the NM-model distinguished the groups in terms of volume (p = 0.048). We also performed intensity thresholding on the atlas_pl masks by discarding voxels below 1.5 standard deviations above the reference in the crus cerebri, and observed a volume divergence between controls and PDs in the expected direction (p = 0.003, all data; p = 0.024, test data). However, this also imposed a strong linear correlation between volume and CNR (r = 0.77, compared to r = -0.07 without thresholding), indicating a high degree of redundancy.

**Fig. 9.**
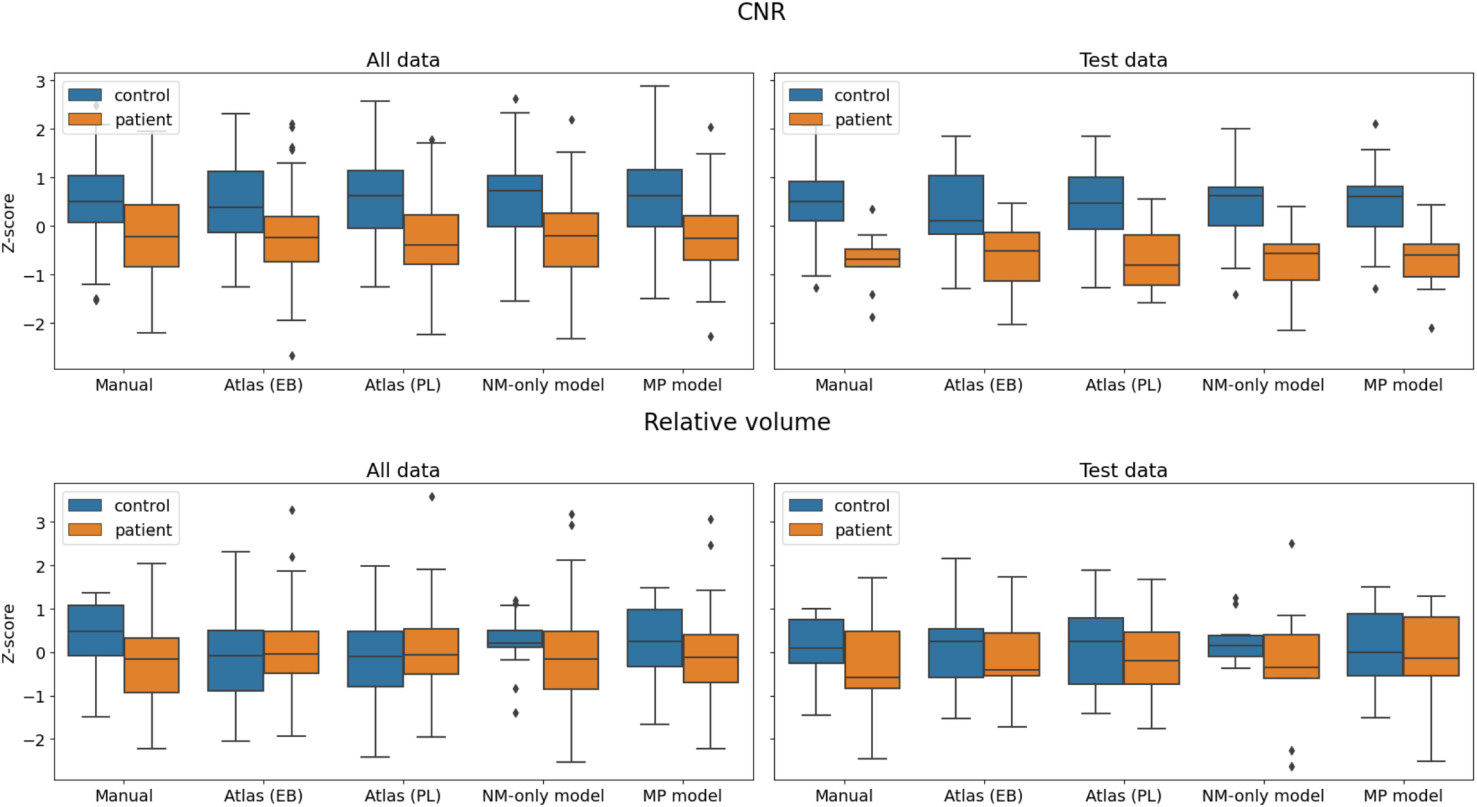
Box and whisker plot for CNR and relative volume for PD versus controls by segmentation method. Boxes showing the inter-quartile range around the median. Top: CNR; Bottom: Relative volume (unthresholded); Left: whole dataset; Right: test set. From left: manual labels; binarized atlas from Biondetti et al. (2020, 2021); our atlas; NM-only model; multiparametric (“all-in-one”) model. Z-normalized for visualization purposes.

The group difference in CNR was much more pronounced than volume, consistently across methods (Fig. 9), with an average 22% signal reduction in patients compared to controls in the test set. The effect size was overall larger in the test group than the whole dataset, but across models the NM content was reliably reduced in PD (13% on average, whole dataset). The strongest effect was within our atlas (p = 0.0004), and none had p-values above 0.01. However, note that atlas_pl was constructed from the full Norwegian dataset, which could inflate its performance. Despite a fair consistency across methods in terms of PD/control difference in CNR, the absolute values were not directly comparable between the atlas-derived values and those from DL or manual labels, with a lower CNR from the atlases, consistent with a lower DSC with the hyperintensity. To facilitate easier comparisons between methods, values were Z-normalized.

**Fig. 10.**
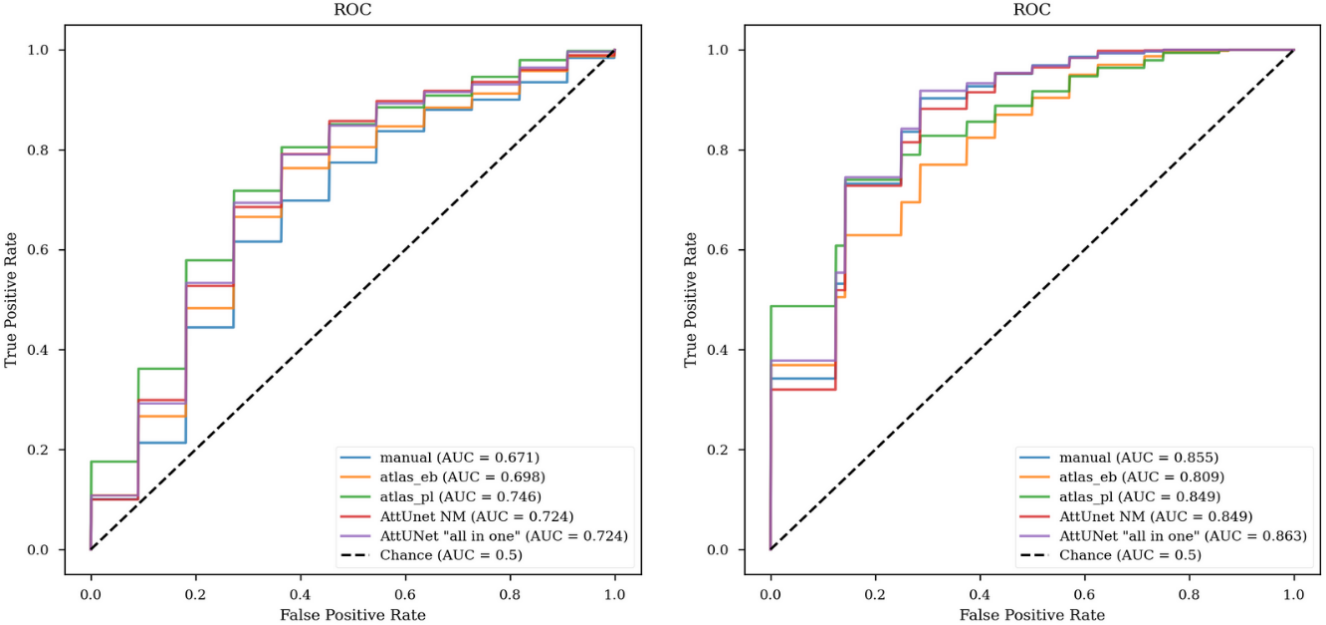
ROC curves for all models in whole dataset (left) and test set (right).

To evaluate discrimination performance from volume and CNR combined, we constructed receiver operator characteristic (ROC) curves with logistic regression and 2-fold CV. Because the area under the curve (AUC) in the test set was highly sensitive to the random seed for the CV splitting due to the moderate sample size, we decided to report the average from 100 seeds (Fig. 10). In the test set, the “all-in-one” model performed best, at 0.863, tightly followed by the manual segmentation (0.855). As we had observed a poorer separation between controls and PDs in the whole dataset (Fig. 9), we repeated the analysis with the pooled data, and noticed a fair drop in AUC, most notable in the manual labels (0.67). Here, the best model was our atlas (0.746), followed by the DL models (both at 0.724). While the estimate for the whole dataset is valid for the manual and atlas-derived masks, the DL models are not independent from the manual labels, and should be interpreted with caution. Excluding the manual labels, the binarized atlas from Biondetti et al. (2021) performed the worst, but note that the authors never intended it to be used in this fashion, but rather as three separate subregions.

#### 3.2.2 Symptomatic laterality

**Fig. 11.**
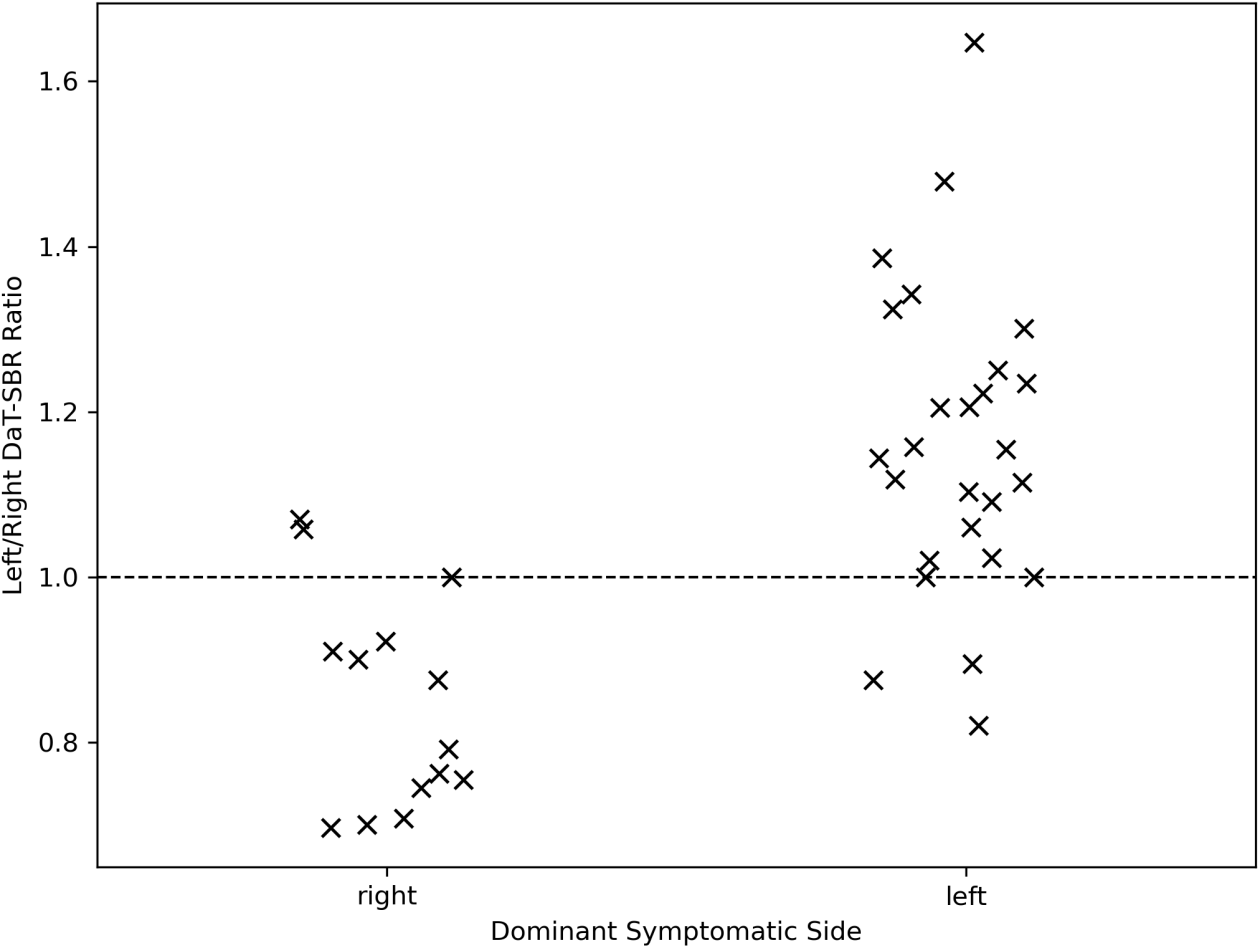
The inter-hemispheric SBR ratio separates left-dominant from right-dominant PD patients.

The SBR from DaT scans shows that nigrostriatal denervation is tightly linked to the contralateral symptom presentation. This was confirmed by computing the ratio of the SBR in the left hemisphere to the right hemisphere (Fig. 11) and performing a Mann-Whitney U test, yielding a $p<10^{-7}$ and a corresponding common language effect size of 0.92, confirming a very good separation. The symptomatic dominant side was defined by taking the sum of brady-rigidity and tremor scores in UPDRS-III for each side, and selecting the side with the highest score. When we repeated the procedure for NM volume, CNR, and magnetic susceptibility, neither were informative of symptom laterality. Furthermore, NM-MRI derived measures correlated poorly to SBR. Only a small correlation between volume and SBR was present, but this ceased after adjusting for total brain volume, suggesting a global atrophy effect.

#### 3.2.3 External clinical validation

**Fig. 12.**
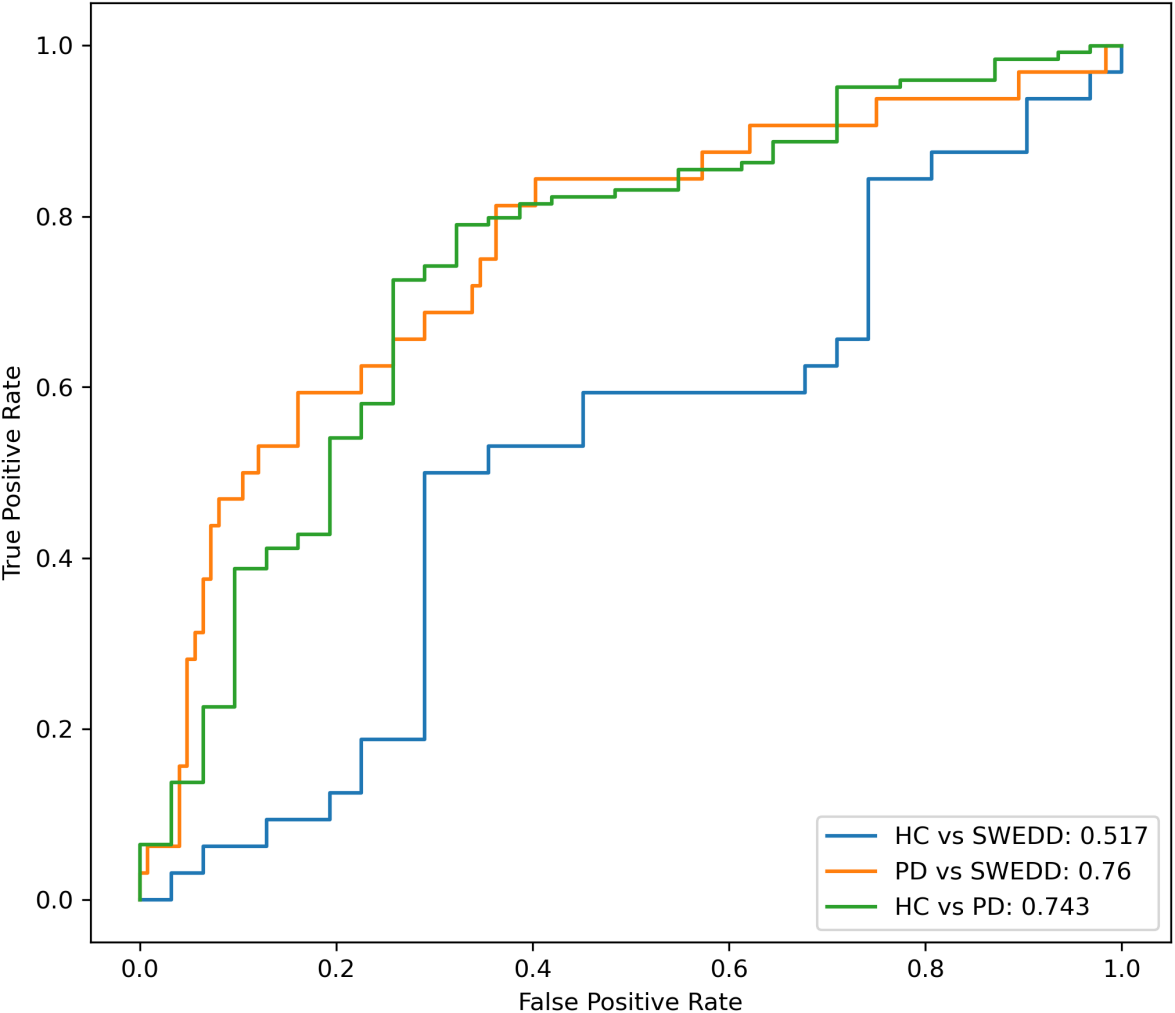
ROC curves from TSE-FS images.

In the end we created a model intended for publishing which was trained on all our available GRE-MT images (N = 131) from all three cohorts, as well as the other contrasts from the Norwegian cohort. This final Attention U-Net was similarly trained for a predefined 50 epochs, and utilizing aggressive augmentation. Since we made use of all the labeled images in training, we evaluated it by means of separating the clinical groups in the 187 unlabeled TSE-FS scans from PPMI. In addition to deriving the CNR, we expanded the analysis of imaging metrics to include the CR between the SN and crus cerebri. Crus cerebri masks were obtained by finetuning the SN segmentation algorithm on the atlas-derived background masks for 10 epochs. We noted an improved group separation with CR compared to CNR, with AUROCs of 0.760 between PD and SWEDD (P = $2.5\times 10^{-6}$ ) and 0.743 between PD and healthy controls (P = $5.2\times 10^{-6}$ ) (Fig. 12). For CNR, the AUROCs were lower, at 0.721 and 0.651 for PD versus SWEDD and PD versus controls, respectively. Neither metric could be used to discern SWEDD from controls.

## 4 Discussion

### 4.1 Multi-contrast segmentation for addressing domain shift

In this work, we have tested various strategies for training a deep neural network to delineate the hyperintensity of the SN from NM-MRI. We utilized both NM-MRI and conventional MRI contrasts, even though sensitivity to NM is only facilitated in the former. Although this approach is slightly counter-intuitive, it did provide a certain degree of regularization. By purposefully training our models only on data from the Norwegian cohort, and validating them on the Canadian and PPMI cohorts, we reproduced the realistic clinical setting of encountering images that do not conform to the expected distribution of the training data, something which is often overlooked in research on medical deep learning applications. The challenge of generalization across sites and scanners—known as domain shift—is one of the major challenges facing the field of deep learning for medical imaging (Kushol et al., 2023; Mårtensson et al., 2020), and is difficult to spot in the absence of explicit scrutiny.

Safeguarding against this type of overfitting is inherently challenging due to the “black box” nature of neural networks, and may result in a model’s decision making to rely in part on features not directly relevant to the task at hand. An instructive example can be drawn from Alzheimer’s disease classification, in which Tinauer et al. demonstrated how convolutional neural networks trained on head MRIs would rely largely on non-brain tissue for making predictions (Tinauer et al., 2022). This example is illustrative, but simultaneously deceptively simple. From subtle intricacies like differences in field inhomogeneity, scanner artifacts, and how images are interpolated, to the choice of pulse sequence, these differences are destined to arise in medical imaging research and practice. This is a well-known problem and has been captured by Andrew Ng: “You take that same model […] to an older hospital down the street, with an older machine, and the technician uses a slightly different imaging protocol, that data drifts to cause the performance of AI system to degrade significantly” (Perry, 2021).

Similarly, we observed severe deterioration in performance from the models that had been trained and tested naively on a single cohort. We tested two strategies for improving out-of-sample predictions: training on multiparametric data and aggressive data augmentation. With the former strategy, in the low augmentation scenario, the results were slightly inconsistent: the robustness was improved in both external validation sets with U-Net, but only in one case with Attention U-Net. The application of aggressive augmentations was considerably more effective, both in terms of magnitude and consistency. The model which combined both strategies demonstrated a slight improvement to using aggressive augmentations alone, although it was a small effect from a limited sample size.

We propose that the theoretical justification for training on multi-contrast data is likely the same as that underlying data augmentation, which is to increase the diversity of the samples while retaining certain invariant features. Despite positive results, the effectiveness of data augmentation was far greater in our experiments. An important difference to consider is that data augmentations can be applied continuously, stochastically, and in composition, thus imposing a unique transformation at every epoch of training, greatly increasing the diversity of the data. Our experimental method, on the other hand, is static in nature. The most crucial drawback to our method is, nonetheless, its “data-hungriness”, and it should therefore only be applied if one has a priori access to a multi-contrast dataset, rather than acquiring data for the purpose of improving model generalization. However, it is simple to implement and requires no specialized architecture or setup for training.

More advanced methods have been developed to exploit the diversity of multimodal images more explicitly for addressing domain shift in computer vision. For instance, Cheng et al. (2025) devised a novel strategy of synthesizing images by combining samples from separate domains (sites) through local or global Fourier transforms (GLFRT), which they used to train a network with a specialized module with dynamic parameters at inference time. Compared to a baseline approach, analogous to ours, their method improved the DSC by 2–4% on three different image datasets. Although their domain was defined in terms of acquisition site, as opposed to contrast type, their findings could suggest that multi-contrast MRIs have a greater potential for domain generalization than we were able to demonstrate in our experiments. Other groups have successfully taken the idea of data augmentations to the extreme by generating synthetic MRI contrasts, using both Gaussian mixture models (Billot et al., 2023; Mauri et al., 2024; Meyer et al., 2021), as well as more physics-informed techniques (Peretti et al., 2023). Most notable is SynthSeg, which is trained on entirely synthetic MRI contrasts, provided segmentation maps only. SynthSeg is reported to achieve excellent performance across a range of common contrasts, although we are not aware of any studies utilizing this approach on NM-sensitive MRI. While our approach is simple to implement, requiring no specialized architecture or setup, its performance could likely benefit from applying similar novelties.

In the canonical multi-channel multiparametric approach, we observed a trend of improved performance compared to single contrast segmentation with U-Net, but the added benefit was small when NM-MRI was one of the included contrasts, and it did not replicate while using Attention U-Net. An additional drawback with this approach is that it presupposes the availability of a particular set of contrasts, without being able to handle incomplete data. We, therefore, deemed it as inconvenient, and found the “all-in-one” approach to be a more seamless, albeit naive, way to utilize multicontrast datasets. A possible weakness to this model is that no explicit information is supplied regarding which contrast is presented, and it is therefore not incentivized to add extra weighting on the NM image as the ground truth. This could possibly be addressed by fine-tuning the model to the NM images after training the base model on the alternative contrasts.

### 4.2 Model performance

Our results showed generally higher values of the DSC metric from the deep learning models than from the atlases. It should be noted that in our atlas labeling, we only used a minimal pipeline with no fine-tuning, while others have built more advanced atlas-labeling methods such as Ariz et al. (2019) and Jin et al. (2022), the latter reporting an excellent DSC with optimized thresholding. In the Norwegian test data, we observed a considerably better segmentation accuracy from the neural networks than from the atlases. However, by focusing our work on validating the models on out-of-distribution cohorts, we arrived at the conclusion that a good performance on independent (in-distribution) test data is no indication of good performance on datasets acquired with different scanner hardware or acquisition parameters. Only after applying suitably heavy augmentations, did the models achieve a higher DSC than the atlases on the external cohorts. The most dramatic discrepancy in the DSC was observed in the low augmentation setting, between the Norwegian test set (DSC = 0.75) and the Canadian cohort (DSC = 0.007). The majority of the gap was closed in the high-augmentation setting (DSC = 0.67). This suggests that the domain shift was driven primarily by image qualities which were embodied by the additional augmentations. We did not investigate the augmentations individually to pinpoint the specific drivers, but considering the strong field inhomogeneity and characteristically different intensity profiles in the Canadian images, we hypothesize that intensity-based augmentations like gamma exponentiation and bias field simulations were decisive. From a study on harmonization strategies for segmenting white matter hyperintensities, bias field removal leads to a 4-fold reduction in the explained variance from the site variable, highlighting the implication of bias fields (Bordin et al., 2021). Some of the remaining performance gap in our model could be explained by an on-average greater atrophy in the Canadian subjects, 91% of whom have PD. After aggressive augmentation, there was still a large divergence in performance between the Norwegian test set and PPMI. In contrast to Canada, this could not be explained by population drift, as we would expect less atrophy from the prodromal subjects who constituted the majority of the cohort.

Looking past segmentation accuracy to clinical performance, did neither of our models outperform the others by a large margin, as judged by the ROC-AUC? Firstly, the “all-in-one” model only had a mild advantage to the atlases in the test set. Secondly, our atlas outperformed the DL models in the pooled (training+test) dataset. However, this should be interpreted with caution as 1) the atlas was constructed from the pooled data, and 2) the DL models’ reduced performances on the training set could be explained by overfitting to the manual labels (notably, having the lowest AUC in the training data). Consequently, we are left with a paradox: although the atlases scored considerably worse in terms of DSC in the test set, this fact did not seem to translate proportionally to clinical utility. One possible explanation could be that the manual labels in our training set simply were of too low quality, which is supported by a poor ROC-AUC in the pooled data. Another, not mutually exclusive possibility is that the accuracy of the segmentation algorithm may not be very crucial to measure group differences, as has been suggested in Le Berre et al. (2019). This might be related to how atrophy is more pronounced in the posterolateral portion of the SN, particularly early in the progression of PD (Biondetti et al., 2020; Lakhani et al., 2024), while our SN labels were traced around the NM hyperintensity, omitting the posterolateral subregion in cases of great atrophy. On the other hand, the deformation field applied to the atlas labels does not warp sufficiently to omit this area, which might be beneficial for capturing atrophy.

### 4.3 Imaging biomarkers

The disease-related CNR reduction in the NM hyperintensity of the SN is well established across studies. Likewise, SNc volume reduction is also commonly reported from studies on NM-MRI (Ariz et al., 2019; Castellanos et al., 2015; Gaurav et al., 2022; Isaias et al., 2016; Kashihara et al., 2011; Pyatigorskaya et al., 2018). While we reproduced the former observation, we observed only a weak trend in terms of volume across the tested methods. Notably, we would not expect atlas-derived labels to capture volumetric loss directly, as this would require an excessive degree of image deformation. A point to consider here is that a fair number of studies (Ariz et al., 2019; Isaias et al., 2016; Jokar et al., 2023; Matsuura et al., 2016; Ogisu et al., 2013; Schwarz et al., 2011; Vitali et al., 2020) incorporate intensity thresholding into the segmentation pipeline. Besides the drawback that an optimal threshold must be empirically set based on pulse sequence and scanner parameters, thresholding has the additional effect of inducing a strong collinearity between signal intensity and volume (r = 0.77 in our data from a threshold at 1.5 standard deviations from background). Collinearity poses a problem because it introduces redundancy between the features, leading to a conflation between volume and signal intensity, raising ambiguity about biological interpretation. It is, nonetheless, an empirical question whether we can achieve better separation by only considering voxels above a certain threshold. In our case however, CNR was a better indicator of disease status than SN volume. In the final evaluation on the TSE-FS scans of PPMI, we made use of both CNR and CR for distinguishing the clinical groups, and observed a better group separation with CR than with CNR. However, as we did not perform this comparative analysis in the former datasets, we cannot generalize this observation. Future studies should evaluate both metrics for determining their relative performance.

### 4.4 Asymmetry

NM-MRI demonstrated a clear inferiority to DaT-imaging in terms of correlation with clinical laterality. Despite our results, studies have been conducted suggesting lateral differentiability from NM-MRI: Matsuura et al. (Matsuura et al., 2016) measured a greater NM hyperintensity loss in the contralateral side in PD from 14 patients. Vitali et al. (2020) reproduced the finding with a larger sample, although it was only reported as a mean group effect and not used to predict on an individual level. Another study with a similar method by (Prasad et al., 2018) reported an accuracy of 61% in predicting the most affected side on a subject basis, but this is hardly better than what can be expected from chance. A likely explanation for the discrepant results is variability in acquisition quality. For instance, the study by Vitali et al. (2020) could also report a greater effect size than us in distinguishing PD and controls. This suggests that a higher sensitivity is required for measuring the laterality effect. On the other hand, the performance is not good enough to conclude that nigrostriatal denervation and somatic degeneration of the SNc, let alone signal loss in the NM hyperintensity, are one-to-one processes.

### 4.5 Conclusion

In this work, we have demonstrated how a seemingly well-performing segmentation model can drop in performance when presented with out-of-distribution data, and explored a potential method to alleviate the problem by incorporating multiparametric MRI into the training. Despite having a positive regularizing effect, this approach could not compete with aggressive data augmentation, although they are not mutually exclusive. Importantly, the optimized models did not outperform the naive models when validated on data from the same site of the training data, so we therefore conclude that external validation is not only helpful but also strictly necessary to close the research-deployment gap. Despite promising initial results, our approach and model will have to be independently evaluated to properly establish its utility versus alternative methods.

## Supplementary Material

Supplementary Material

## Data Availability

Code for accessing and applying the segmentation model with a curated Jupyter notebook is available from https://github.com/lillepeder/snceg.
