# Supplementary material for: Deep-learning segmentation of the substantia nigra from multiparametric MRI: Application to Parkinson’s disease
Source: Imaging Neurosci (Camb). 2025 Sep 29;3:IMAG.a.158. doi: 10.1162/IMAG.a.158 (PMC12479381; doi:10.1162/IMAG.a.158)
Supplement: Supplementary Material [file IMAG.a.158_supp.pdf]

Supplementary Material

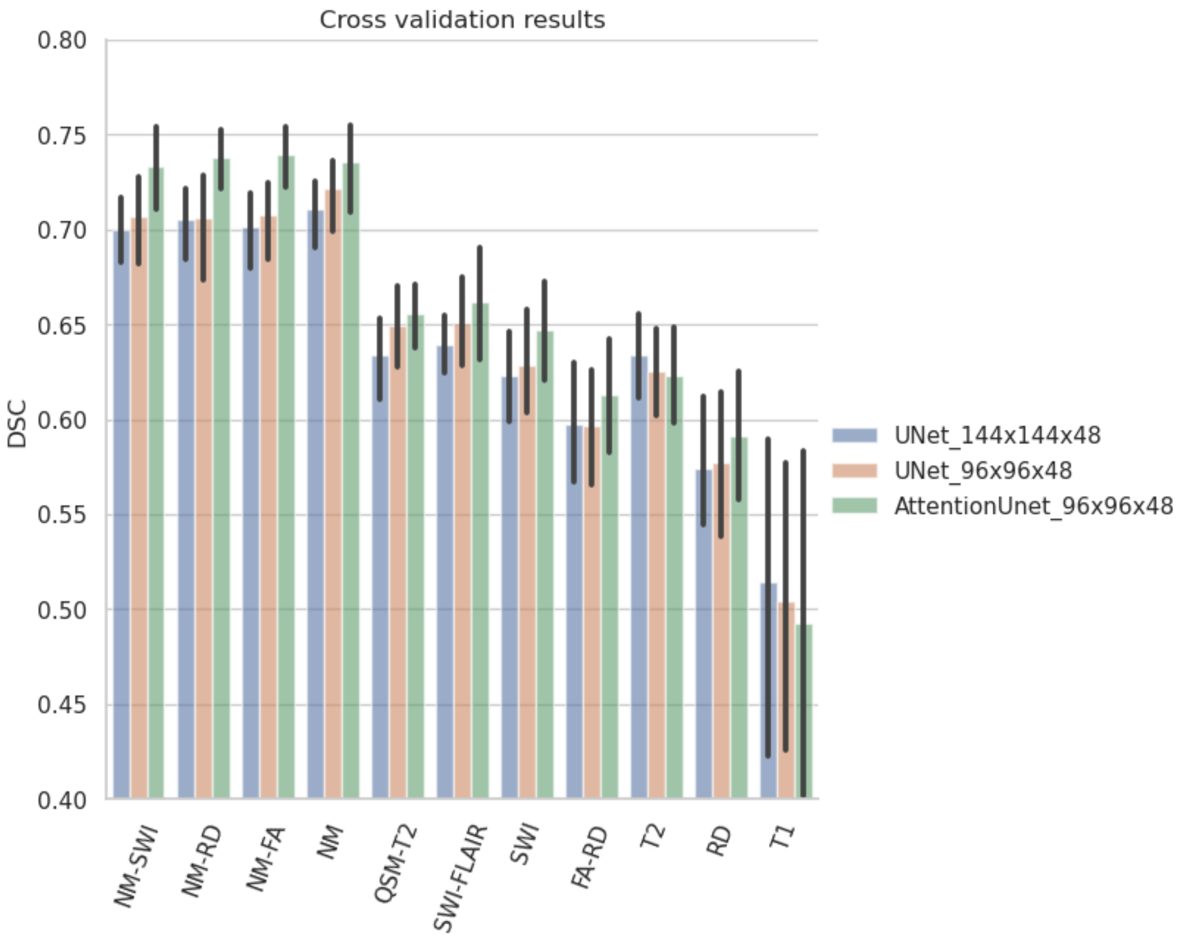

Figure S1: DSC from 6-fold cross validation on the training set, U-Net and Attention U-Net. U-Net was tested at two image sizes (144x144x48 and 96x96x48), and Attention U-Net could only be tested on the smaller size. The set of contrasts the model was trained and validated on is indicated on the x-axis. Error bars in black (+/- standard deviation).

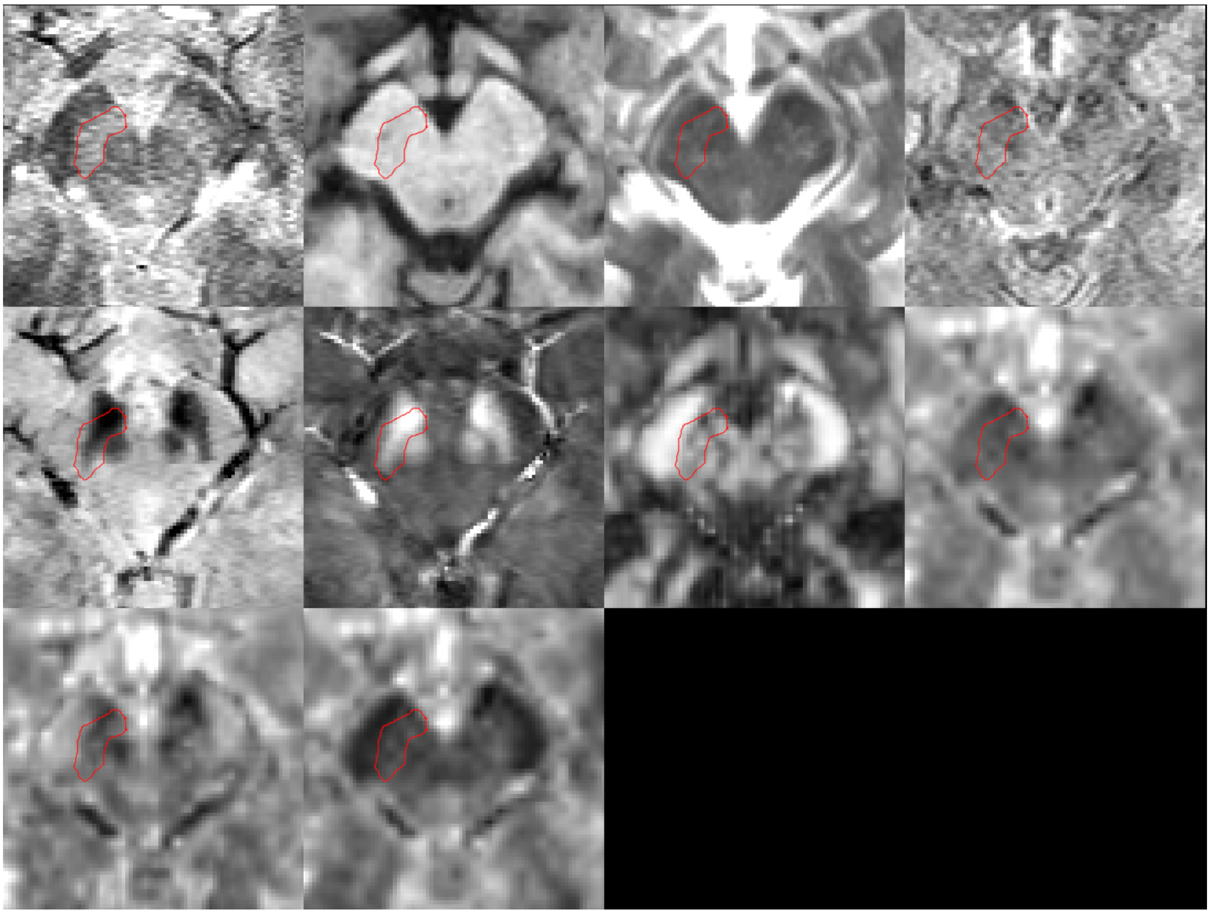

Figure S2: Midbrain (axial slice) of PD patient, showing all MRI contrasts used in the study. The right NM-hyperintensity of the SN is delineated in red for reference. The scans were coregistered and resampled to the NM image. Top row, from left: NM-MRI, T1w, T2w, T2-FLAIR. Middle row, from left: tSWI, QSM, FA, MD. Bottom row, from left: AD, RD.

Table S1: DSC of the all-in-one model (Attention U-Net), predictions on all contrasts (Norwegian test set, low augmentation setting).

**Contrast**	**DSC**
**NM**	0.746
**tSWI**	0.690
**FLAIR**	0.676
**QSM**	0.663
**T2**	0.658
**MD**	0.653

**RD**	0.620
**T1**	0.610
**FA**	0.584
**AD**	0.516

Model specifications

All models were trained on a batch size of 4, and convolutional kernel size of 3x3x3. U-Net was always trained with 5 layers of shape (16, 32, 64, 128, 256), with stride length of two, and two residual units. Attention U-Net were built with 4 layers of shape (32,64,128,256).

Images were resampled to the default resolution: 0.67, 0.67, 1.34 mm^3. In the multi-contrast screening, images were cropped to (96,96,48) in the input layer. In the cross validation, the input images were cropped to size (144,144,48) or (96,96,48) for U-Net. Attention U-Net were trained on a smaller crop size (96,96,48) to account for hardware limitations. The smaller size was used for all remaining analyses.

In the low-augmentation setting, the following augmentations were applied randomly with a 50 percent probability: left-right mirroring, and affine transforms comprising +/- 10% scaling, 5 degrees of rotation, translation (up to 5 voxels) in the xy-plane, with cubic b-spline interpolation. For the aggressive augmentations, the list was expanded: Affine augmentations were increased to +/- 20% size, 20 degrees of rotation, and 10 voxels of translation in all directions. We further added RandomGamma, RandomBiasField, RandomMotion, RandomNoise and RandomElasticDeformation from TorchIO.

For the semi-automatic labelling of the training data, a U-Net was trained (with the same specifications as provided above, and the low augmentation setting) on the 31 manually labelled NM-scans for 100 epochs, to predict the remaining 25 training labels. Manual corrections were applied.
